# Ryanodine receptor RyR1-mediated elevation of Ca^2+^ concentration is required for the late stage of myogenic differentiation and fusion

**DOI:** 10.1186/s40104-021-00668-x

**Published:** 2022-02-11

**Authors:** Kai Qiu, Yubo Wang, Doudou Xu, Linjuan He, Xin Zhang, Enfa Yan, Lu Wang, Jingdong Yin

**Affiliations:** 1 State Key Laboratory of Animal Nutrition, College of Animal Science and Technology, China Agricultural University, Beijing, 100193 China; 2 Risk Assessment Laboratory of Feed Derived Factors to Animal Product Quality Safety of Ministry of Agriculture & Rural Affairs & National Engineering Research Center of Biological Feed, Institute of Feed Research, Chinese Academy of Agricultural Sciences, Beijing, 100081 China

**Keywords:** Apoptosis, Ca^2+^ homeostasis, Endoplasmic reticulum stress, Myoblast fusion, Myogenic differentiation, RyR1 knockout

## Abstract

**Background:**

Cytosolic Ca^2+^ plays vital roles in myogenesis and muscle development. As a major Ca^2+^ release channel of endoplasmic reticulum (ER), ryanodine receptor 1 (RyR1) key mutations are main causes of severe congenital myopathies. The role of RyR1 in myogenic differentiation has attracted intense research interest but remains unclear.

**Results:**

In the present study, both RyR1-knockdown myoblasts and CRISPR/Cas9-based RyR1-knockout myoblasts were employed to explore the role of RyR1 in myogenic differentiation, myotube formation as well as the potential mechanism of RyR1-related myopathies. We observed that RyR1 expression was dramatically increased during the late stage of myogenic differentiation, accompanied by significantly elevated cytoplasmic Ca^2+^ concentration. Inhibition of RyR1 by siRNA-mediated knockdown or chemical inhibitor, dantrolene, significantly reduced cytosolic Ca^2+^ and blocked multinucleated myotube formation. The elevation of cytoplasmic Ca^2+^ concentration can effectively relieve myogenic differentiation stagnation by RyR1 inhibition, demonstrating that RyR1 modulates myogenic differentiation via regulation of Ca^2+^ release channel. However, RyR1-knockout-induced Ca^2+^ leakage led to the severe ER stress and excessive unfolded protein response, and drove myoblasts into apoptosis.

**Conclusions:**

Therefore, we concluded that Ca^2+^ release mediated by dramatic increase in RyR1 expression is required for the late stage of myogenic differentiation and fusion. This study contributes to a novel understanding of the role of RyR1 in myogenic differentiation and related congenital myopathies, and provides a potential target for regulation of muscle characteristics and meat quality.

**Supplementary Information:**

The online version contains supplementary material available at 10.1186/s40104-021-00668-x.

## Introduction

Ryanodine receptor 1 (RyR1), located on the endoplasmic/sarcoplasmic reticulum (ER/SR) membrane, is highly expressed in the skeletal muscle. It serves as a critical Ca^2+^ channel in mediating intracellular flux of Ca^2+^, triggering the contraction of the skeletal muscle. Various mutations or epigenetic changes in the *RyR1* gene have been demonstrated to associate with muscle myopathies including malignant hyperthermia and several congenital myopathies [1–4]. RyR1 crystal structure has been resolved using electron cryomicroscopy as a 6-transmembrane ion channel with an EF-hand domain for Ca^2+^-mediated allosteric gating and a huge cytoplasmic domain on top of each transmembrane domain [5–7]. These studies contribute to the deep understanding of the structure, function and channel activity of RyR1. In particular, the impacts of two malignant hyperthermia-associated mutations on the RyR1 3D structure were recently solved to gain insights into the pathogenesis [8, 9]. Diagnostic gene-sequencing has been employed to avoid RyR1-related congenital myopathies, and several disease-modulating therapeutic strategies and salvage therapies have been developed against RyR1-related myopathies [10–15].

It has been well known that cytoplasmic Ca^2+^ mediates myogenic differentiation and skeletal muscle development [16]. As a major Ca^2+^ release channel of endoplasmic reticulum (ER), RyRs-mediated Ca^2+^ release plays a role in the morphogenesis of mammalian skeletal muscle, while most of RyR1 mutations present gain-of-function phenotype and result in leaking of the internal Ca^2+^ store [17]. Notably, the proportion of pigs carrying the single-base mutation of RyR1 unexpectedly increased during intensive breeding for lean meat in pigs, which leads to the increased yield of abnormal meat characteristics of pale, soft and exudative [18]. However, the block of RyRs activity by ryanodine selectively retards fetal myoblast differentiation, and lead to both physiological and pathological consequences of muscle morphology and functions [19]. It has been demonstrated that homozygous RyR1-null mice died after birth and displayed small limbs and abnormal skeletal muscle organization [20, 21]. Therefore, we hypothesize that RyR1 is not only involved in causing congenital myopathies, but implicated in myogenesis and subsequent muscle development. Therefore, the mechanisms of RyR1 action in myogenesis need to be elucidated.

Endoplasmic/sarcoplasmic reticulum is responsible for proper folding, processing, and trafficking of proteins and plays an important role in cellular Ca^2+^ homeostasis. Alterations in cellular Ca^2+^ dynamics directly trigger ER stress and activate the unfolded protein response (UPR) [22, 23]. ER stress and resultant UPR modulation are implicated in various human diseases including sarcopenia [24–26], and roles of ER stress and UPR pathways in skeletal muscle health and diseases receive increased research attention [27]. Therefore, we surmise that ER stress signaling is involved in RyR1-related muscle myopathies.

Myogenic differentiation and fusion play a pivotal role in the development and skeletal muscle maturation via forming mature multinuclear myofibers, impacting on meat quality in livestock [28]. In this study, we employed CRISPR/Cas9-based RyR1-KO myoblasts and siRNA-mediated RyR1-knockdown myoblasts to explore the role of RyR1 in myogenesis and formation mechanism of RyR1-related myopathies.

## Materials and methods

### Cell culture and myogenic differentiation

Mouse skeletal myoblast C2C12 cells were purchased from the National Infrastructure of Cell Line Resource in China. Proliferating myoblasts were maintained in DMEM/high glucose medium (Hyclone, Logan, UT, USA) supplemented with 10% FBS (Gibco, Carlsbad, CA, USA) in a humidified CO_2_ incubator (5% CO_2_, 37 °C; HF90, Heal Force, Hongkong, China). For myogenic differentiation, myoblasts with 80% ~ 90% confluence were induced by DMEM/high glucose medium containing 2% horse serum (Hyclone, Logan, UT, USA).

Myogenic cells of pigs (*n* = 3) were isolated using preplate techniques from the skeletal muscle according to the protocol our lab established previously [29, 30]. The cells were cultured in growth medium in a 100 mm dish coated with collagen I (Sigma-Aldrich, Louis, MO, USA) at 37 °C and 5% CO_2_. The growth medium was composed of DMEM/F12 (Hyclone), 10% FBS (Gibco-BRL, Carlsbad, CA, USA), 2 mmol/L glutamine (Gibco-BRL), and 5 ng/mL bFGF (Peptech, Burlington, MA, USA). As for myogenic induction, cells were cultured for 5 d in DMEM/F12 medium containing 2% horse serum (Hyclone).

### Cellular Ca^2+^ concentration measurement

Ca^2+^ concentration in the cytoplasm or ER was measured using flow cytometry. Briefly, cells were collected, washed with PBS (phosphate buffered saline) and HBSS (Hanks balanced salt solutions) subsequently, and then incubated in 5 μg/mL Fluo-3 acetoxymethyl ester (Cayman, Ann Arbor, MI, USA) or Mag-fluo-AM (GENMED, Shanghai, China) for 30 min at 37 °C in dark. After three washes with PBS supplemented with 1% FBS, cells were resuspended in 200 μL PBS containing 1% FBS. Flow cytometry was carried out immediately using a FACS Calibur Cytometer and Image Cytometry software (BD, Franklin, NJ, USA). Calcium-bound Fluo-3 or Mag-fluo-AM has an emission maximum of 526 nm which was quantified by excitation with a 488-nm laser and signals were collected using a 530/30 nm band-pass filter. Each sample generated 20,000 live gated events. Debris, multicellularity, and dead cells were excluded by forward scatter (FSC) and side scatter. For detecting dynamic change of Ca^2+^ concentration of unfused cells during myogenic differentiation, a blank control combined with a house-keeping control (the proliferative C2C12 cells) was used to correct the deviation caused by the loaded indicator amount and the voltage used in each measurement. Mean fluorescence intensity was determined from the entire cell population and then adjusted by relative cell size calculated according to FSC to represent Ca^2+^ concentration.

### Cell viability and apoptosis assays

Cell viability was measured using Cell Counting Kit-8 (CA1210, Solarbio, Beijing, China) according to the manufacturer’s guideline. Briefly, cells were cultured in 96-well plates for 24 h, and then CCK-8 reagent was added at 100 μL per well. One hour later, the absorbance of culture medium was analyzed by microplate spectrophotometer. In addition, cell proliferation activity was also measured by Cell-Light™ EdU Apollo®488 Cell Tracking Kit (RIBOBIO, Guangzhou, China). After pre-cultured for 24 h in 96-well plates, cells were cultured continuously for another 2 h in new media supplemented with 50 μmol/L EdU reagent. Then cells were fixed by 4% paraformaldehyde, permeabilized by 0.2% Trutib X-100, and fluorescently-tagged with Hoechst3342 using nucleus staining methods. The newly proliferated cells were visualized by an Apollo reaction system. Cell proliferation rate was analyzed by ImageJ (v1.51h, National Institutes of Health, Bethesda, MD, USA).

Apoptosis was tested using an Annexin V-FITC/PI Apoptosis Detection Kit (Gene Protein Link, Beijing, China) according to the manufacturer’s protocol. Briefly, cells were stained with a combination of Annexin V-FITC and propidium iodide in darkness for 15 min at room temperature, and then analyzed by the flow cytometry system.

### RNA isolation and qRT-PCR

Cells used for total RNA extraction obtained from 3 separate experiments (different batches of cells and on different days). Total RNA was extracted from cells using HiPure Total RNA Mini Kit (Magen, Beijing, China), and then reverse-transcribed into cDNA using a PrimeScript™ RT reagent Kit with gDNA Eraser (Takara, Osaka, Japan). Synthesized cDNA was used for RT-qPCR analysis by employing a quantitative real-time PCR kit (Takara, Osaka, Japan) with an AJ qTOWER 2.2 Real-Time PCR system (Analytik Jena AG, Jena, Germany) according to standard procedures. All samples were measured in triplicate. The primers used in the experiment were listed in Table S1. To compare the mRNA expression of *RyR1* and *RyR3* in cells, the amplification efficiency of their primers was used to rectify the qRT-PCR cycle number. *GAPDH* (glyceraldehyde-3-phosphate dehydrogenase) was used as an internal control. Relative gene expression level was calculated by 2^−ΔΔCt^ method [31].

### Protein extraction and western blot analysis

The relative abundances of proteins concerning ER stress, MAPK signaling pathway, and apoptosis were determined by Western Blot. Cell samples were collected and lysed in RIPA buffer (Huaxingbio, Beijing, China) composed of 50 mmol/L Tris-HCl (pH 7.4), 150 mmol/L NaCl, 1% NP-40, and 0.1% SDS, plus a Halt protease/phosphatase inhibitor cocktail (Thermo Fisher Scientific, Waltham, MA, USA). The homogenate was centrifuged at 14,000 × *g* for 15 min at 4 °C and the supernatant was isolated for Western Blot analysis. Protein concentrations were determined using a BCA Protein Assay Kit (Huaxingbio, Beijing, China). Equal amounts of protein (30 μg), together with a pre-stained protein ladder (Thermo Fisher Scientific, Waltham, MA, USA), were electrophoresed on SDS polyacrylamide gel, electro- transferred to a polyvinylidene difluoride membrane (Millipore, Bedford, OH, USA), and blocked for 1 h in 5% non-fat dry milk at room temperature in Tris-Buffered saline and Tween-20 (TBST; 20 mmol/L Tris-Cl, 150 mmol/L NaCl, 0.05% Tween 20, pH 7.4). Samples were incubated with corresponding primary antibodies overnight at 4 °C. After washing with TBST (pH 7.4), membranes were incubated with the secondary antibody (DyLight 800, Goat Anti-Rabbit IgG). Protein bands were detected with the Odyssey Clx kit (LI-COR, Lincoln, NE, USA) and quantified using an Alpha Imager 2200 (Alpha InnoTec, CA, USA). GAPDH was taken as an internal standard to calculate relative protein expression. Antibodies used for Western Blot in this study were listed in Table S2.

### Immunocytochemistry

Cells were fixed with 4% paraformaldehyde (PFA)/PBS for 30 min. After the neutralization of excess formyl group by 2 mg/mL glycine, cells were permeabilized by 0.2% Trutib X-100 in PBS for 10 min. After blocked with 3% BSA/PBS, cells were incubated with primary antibody (anti-RyR1, 1:300, MA3–925, Thermo Fisher Scientific, Waltham, MA, IL, USA; anti-myosin, 1:300, M4276, Sigma-Aldrich, Louis, MO, USA) overnight and then incubated with Fluorescein-Conjugated secondary antibody (ZF-0311, ZSGB-BIO, Beijing, China) at 1:100. Nuclei were stained with DAPI (Thermo Fisher Scientific, Waltham, MA, USA). Finally, myotubes were visualized by an inverted fluorescence microscope.

### Chemical blockers of Ca^2+^ channels

Two kinds of chemical blocker were employed in the experimental treatments. Dantrolene (DAN) (Stock solution: 200 mmol/L in DMSO, HY-12542A, MedChemExpress, South Brunswick, NJ, USA), an inhibitor of RyR1, was added as a final concentration of 10 μmol/L in culture media. Thapsigargin (THA, ab120286, Abcam, Cambridge, UK), an ER stress-inducing agent, was used at a final concentration of 100 nmol/L in culture media. Equal amounts of vehicle (DMSO) were used as the control. During the proliferating period, myoblasts were treated with chemical blockers, respectively, for 48 h and then collected for further analysis. Upon myogenic induction, myoblasts were treated with chemical blockers, respectively, for 5 d and then used for mRNA extraction and immunocytochemistry. Each treatment was conducted in three independently repeated experiments.

### Small interfering RNA transfection

RNA interference of *RyR1* (mouse, gene ID: 20190) expression was performed using a 21-base pair small interfering RNA (siRNA) duplex (designed and synthesized by IBSBIO, Shanghai, China). The sense strand nucleotide sequence for *RyR1* siRNA was 5′-CCUGCUCUAUGAACUUCUAGC-3′ (sense strand) and 5′-UAGAAGUUCAUAGAGCAGGUU-3′ (anti-sense strand). A scrambled siRNA (siControl, sense strand: 5′-UUCUCCGAACGUGUCACGUTT-3′, anti-sense strand: 5′-ACGUGACACGUUCGGAGAATT-3′) with the same nucleotide composition as *RyR1* siRNA but lacks significant sequence homology to the *RyR1* was also designed as a negative control. Briefly, myoblasts were plated in a 100 mm cell culture dish for 24 h, and then transfected with 100 nmol/L siRNA using 16 μL Lipofectamine 3000 (Invitrogen, Carlsbad, CA, USA) in each dish. After transfection for 24 h, myogenic differentiation was induced in cells.

### CRISPR/Cas9 gene-editing

Gene-edited myoblasts with RyR1-knockout were generated via the Clustered Regularly Interspaced Short Palindromic Repeats (CRISPR)-CRISPR associated protein 9 (Cas9) system. The plasmid vectors expressing Cas9 protein and guide-RNA (gRNA) were designed and synthesized by Syngentech (Beijing, China). One gRNA among the three gRNA targeting *RyR1* was selected for using in further study according to their shearing efficiency. The sequences of gRNA-1, gRNA-2, and gRNA-3 are as follows: 5′-GGCGATGATCTCTATTCTTA-3′, 5′-TACAGCCCCTACCCCGGAGG-3′, and 5′-AGCTCAGGCCACCCACCTGA-3′, respectively. When the myoblasts were grown to 80% ~ 90% confluency, 1 × 10^6^ cells were collected and transfected with 2 μg CRISPR-Cas9 vectors by electroporation using Nucleofector Program B-032 (VCA-1003, Lonza, Basel, Switzerland). Infected myoblasts were selected by incubation with 200 μg/mL hygromycin for 1 week. The stable RyR1-knockout cell line was obtained through single cell clone techniques. Genotype identification and verification of putative off-target sites (Table S3) were conducted via DNA-sequencing technology. The related primers were list in Table S4.

### Statistical analysis

Student’s t-test was used between two groups; one-way or two-way ANOVA with Tukey’s test was used among multiple groups. Data are presented as mean ± SEM. The criterion for statistical significance was set at *P* < 0.05.

## Results

### Cytoplasmic Ca^2+^ dynamics and expression patterns of Ca^2+^ channels during myogenic differentiation

**Fig. 1:**
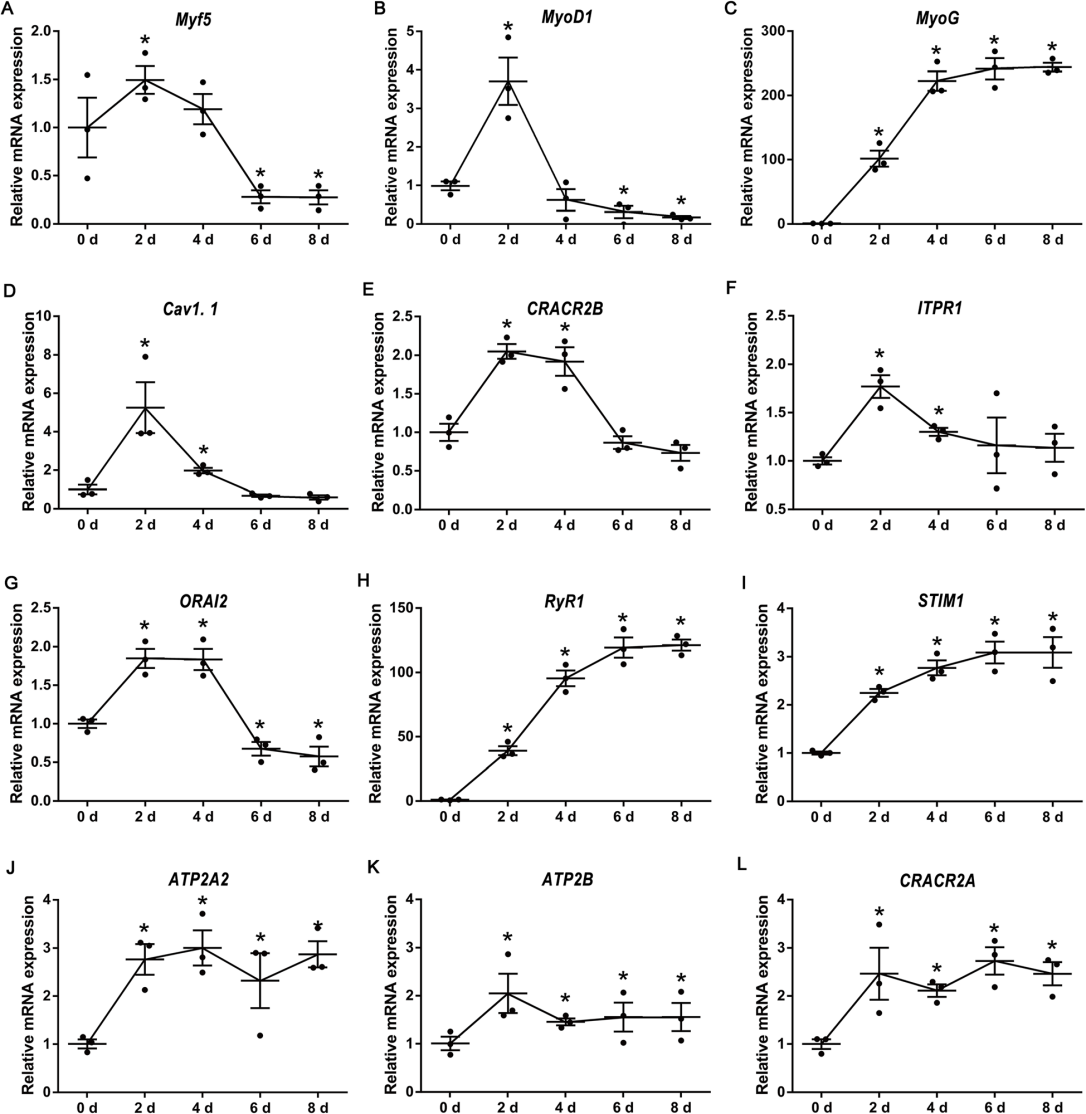
Relative mRNA expression of *Myf5* **(A)**, *MyoD1* **(B)**, *MyoG* **(C)**, *CAV1.1* **(D)**, *CRACR2B* **(E)**, *ITPR1* **(F)**, *ORAI2* **(G)**, *RyR1* **(H)**, *STIM1* **(I)** *ATP2A2* **(J)**, *ATP2B* **(K)**, and *CRACR2A* **(L)**, on d 0, 2, 4, 6, and 8 during myogenic differentiation of C2C12 cells (*n* = 3). The data are presented as the mean ± SEM. *Represents significant difference with the value on d 0 (*P* < 0.05)

Upon myogenic induction, myoblasts gradually expressed plenty of myosin from the d 0 to 6 (Fig. S1A). During the entire period (d 1–5) of myogenic differentiation, cytoplasmic Ca^2+^ concentration (labeled by Fluo-3) of myoblasts was significantly increased (Fig. S1B and C). *Myf5* (myogenic factor 5) and *MyoD1* (myogenic differentiation 1) expression were significantly increased on d 2 relative to d 0, then sharply decreased on d 6 and 8 and even lower than the initial level before myogenic induction (Fig. 1A and B). Meanwhile, *MyoG* (myogenin) expression showed continuous increase and reached a plateau on d 4 (Fig. 1C).

As for Ca^2+^ transporters, *CAV1.1* (also known as *CACNA1S*, calcium voltage-gated channel subunit alpha1 S), *CRACR2B* (calcium release activated channel regulator 2B), *ITPR1* (inositol 1,4,5-trisphosphate receptor type 1), and *ORAI2* (ORAI calcium release-activated calcium modulator 2) whose expression patterns showed similar with *Myf5* and *MyoD1* (Fig. 1D-G). The expression patterns of *RyR1* and *STIM1* (stromal interaction molecule 1) resembled *MyoG* well (Fig. 1H and I). Notably, *RyR1* mRNA expression increased more than 100-fold in myoblastic C2C12 cells upon myogenic induction. Consistently, RyR1 protein expression was significantly increased upon myogenic induction (Fig. S1D and E). In addition, the mRNA expression of *ATP2A2* (ATPase ER/SR Ca^2+^ transporting 2; Fig. 1J), *ATP2B* (ATPase plasma membrane Ca^2+^ transporting 1; Fig. 1K) and *CRACR2A* (calcium release activated channel regulator 2A; Fig. 1L) were significantly increased in myoblastic C2C12 cells on d 2 during myogenic induction and maintained a plateau for subsequent days.

Since *RyR3* mRNA expression level was less than 1/10 that of *RyR1* in C2C12 cells, the increase in *RyR3* mRNA expression level during myogenic induction was far below that of *RyR1* (Fig. S2A and B). From this view, therefore, *RyR1* rather than *RyR3*, another member of *RYRs* expressed in skeletal muscle, may exert a major role in mediating myogenesis of myoblastic C2C12 cells can be attributed to. Consistently, we also observed that *RyR1* mRNA expression showed almost a 25-fold increase during myogenic differentiation, while the mRNA expression of *RyR3* was not changed in myogenic cells of pigs (Fig. S2C-E).

### RyR1-mediated elevation of cytoplasmic Ca^2+^ concentration is indispensable for myogenesis

**Fig. 2:**
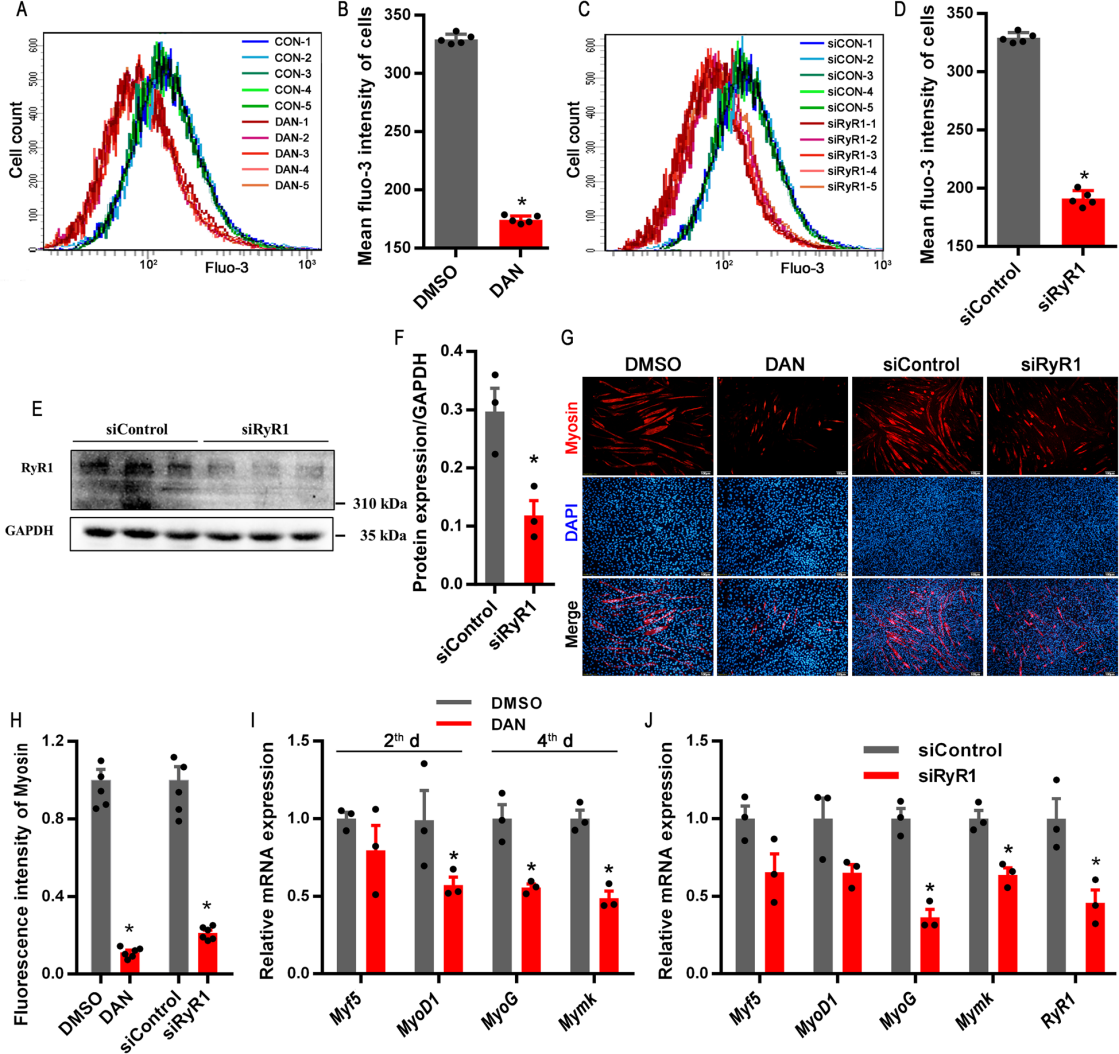
The effects of RyR1 inhibition on myogenic differentiation of C2C12 myoblasts. **(A-B)** The concentration of cytoplasmic Ca^2+^ labeled by Fluo-3 treated with Dantrolene (DAN, 10 μmol/L, RyR1 inhibitor) for 48 h (*n* = 5). **(C-D)** The concentration of cytoplasmic Ca^2+^ labeled by Fluo-3 in C2C12 cells transfected with siRyR1 for 72 h (*n* = 5). **(E-F)** The proteins expression of RyR1 in C2C12 cells after myogenic induction for 5 d with siRyR1 transfection (*n* = 3). **(G-H)** Immunostaining with myosin antibody of C2C12 cells after myogenic induction for 5 d with DAN treatment or siRyR1 transfection (*n* = 6). **(I)** Relative mRNA expression of related genes in C2C12 cells treated with DAN on d 2 (*Myf5* and *MyoD1*) and 4 (*MyoG* and *Mymk*) of myogenic differentiation (*n* = 3). **(J)** Relative mRNA expression of *RyR1*, *Myf5*, *MyoD1*, *MyoG*, and *Mymk* in C2C12 cells transfected with siRyR1 for 72 h (*n* = 3). *Represents significant difference between the two groups (*P* < 0.05)

As shown in Fig. 2A and B, cytoplasmic Ca^2+^ concentration was significantly decreased upon treatment with dantrolene (DAN), an inhibitor of RyR1, which blocks the release of Ca^2+^ from SR/ER [32]. Moreover, RyR1 knockdown by siRNA significantly also decreased cytoplasmic Ca^2+^ concentration of C2C12 cells (Fig. 2C and D). Consistently, the protein expression of RyR1 was effectively suppressed by siRNA interference (Fig. 2E and F). Functional constraints of RyR1 by either DAN or siRyR1 dramatically inhibited the formation of multinucleated myotubes (Fig. 2G and H). During myogenic induction, DAN effectively blocked *MyoD1* expression on d 2 and both *MyoG* and *Mymk* (myomaker, myoblast fusion factor) expression on d 4 (Fig. 2I). At the d 4 during myogenic induction, siRyR1-knockdown significantly reduced *RyR1*, *MyoG*, and *Mymk* expression without impact on *Myf5* and *MyoD1* expression (Fig. 2J).

**Fig. 3:**
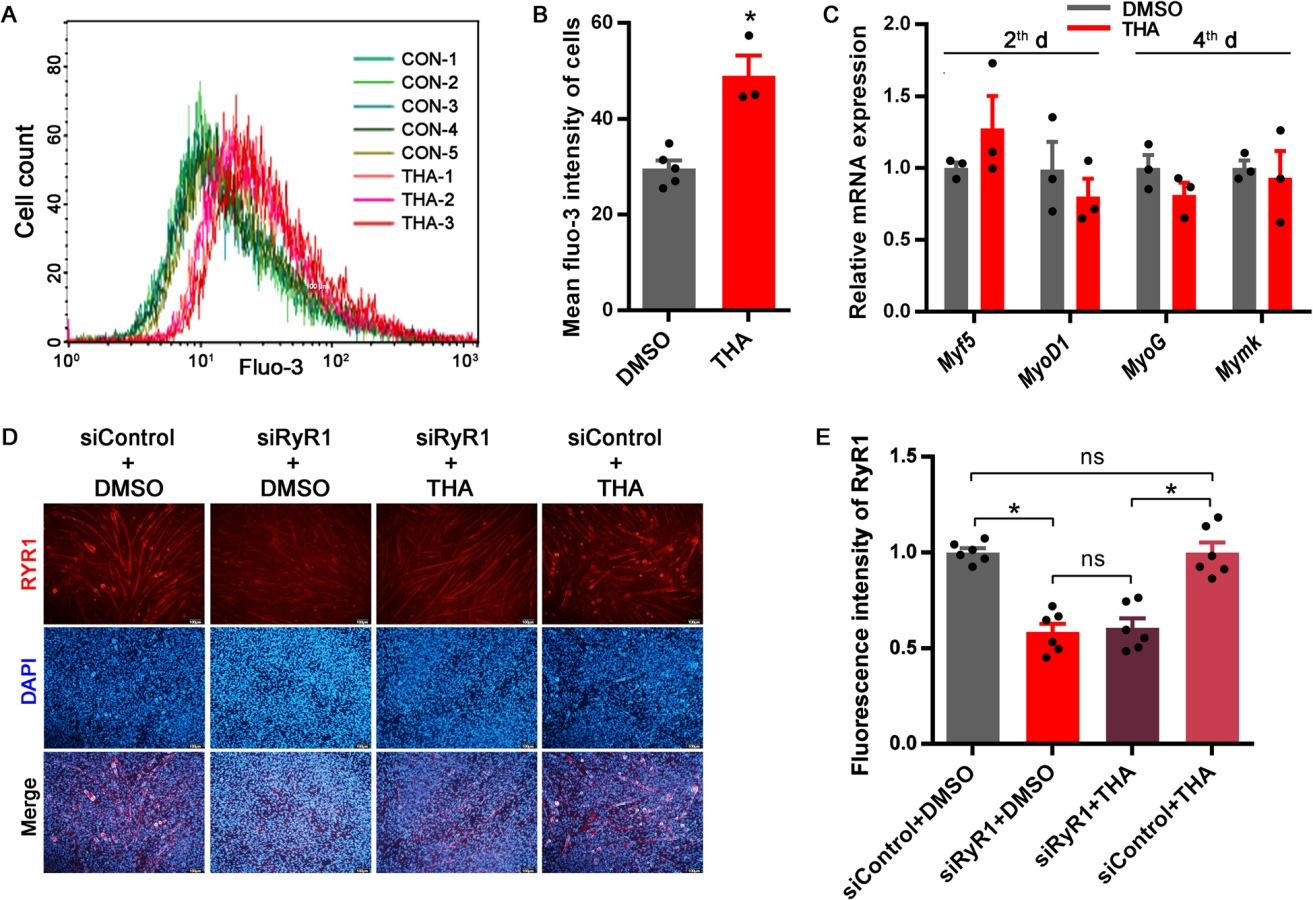
THA-increased cytoplasmic Ca^2+^ concentration was independent of RyR1. **(A-B)** The concentration of cytoplasmic Ca^2+^ labeled by Fluo-3 in C2C12 cells treated with thapsigargin (THA) for 48 h (*n* = 3). **(C)** Relative mRNA expression of related genes in C2C12 cells treated with THA on d 2 (*Myf5* and *MyoD1*) and 4 (*MyoG* and *Mymk*) during myogenic differentiation (*n* = 3). **(D-E)** Immunostaining with RyR1 antibody of C2C12 cells after myogenic induction for 5 d treated with siRyR1 or THA (*n* = 5). The data are presented as the mean ± SEM. *Represents significant difference between the two groups (*P* < 0.05)

**Fig. 4:**
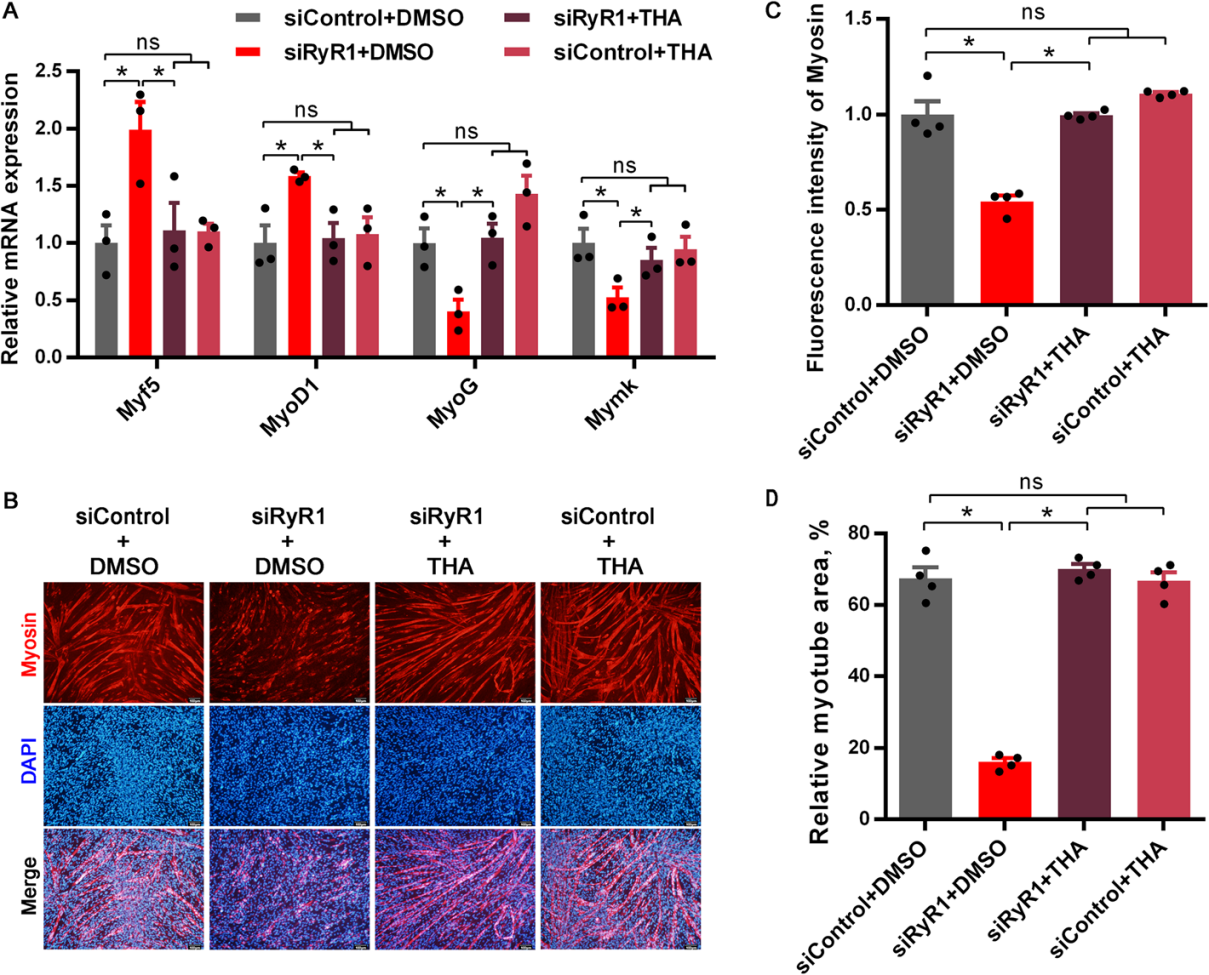
The elevation of cytoplasmic Ca^2+^ concentration is required for myoblast fusion into myotubes. **(A)** Relative mRNA expression of *Myf5*, *MyoD1*, *MyoG*, and *Mymk* after the treatments with siRyR1 or thapsigargin (THA) after myogenic induction for 4 d (*n* = 3). **(B-C)** Immunostaining with myosin antibody after myogenic induction for 5 d treated with siRyR1 or THA (*n* = 4). **(D)** The relative myotube area in **B**. The data are presented as the mean ± SEM. *Represents significant difference between the two groups (*P* < 0.05)

Thapsigargin (THA) treatment significantly increased cytoplasmic Ca^2+^ concentration (Fig. 3A and B), but did not influence the mRNA expressions of myogenic-specific genes including *Myf5*, *MyoD1*, *MyoG* and *Mymk* (Fig. 3C). RyR1-knockdown by siRNA interference was independent of THA treatment (*P* < 0.01 for siRNA treatment, Fig. 3D and E). On d 4 during myogenic induction, siRyR1-knockdown significantly increased *Myf5* and *MyoD1* mRNA expression, while THA effectively eliminated alterations in *Myf5* and *MyoD1* mRNA expressions induced by siRyR1-knockdown (*P* < 0.05 for siRNA treatment × THA treatment, Fig. 4A). Accordingly, myotube formation was significantly inhibited by siRyR1-knockdown, which was effectively recovered by THA (*P* < 0.01 for siRNA treatment × THA treatment, Fig. 4B-D).

### Effects of RyR1-knockout on cell proliferation and differentiation

**Fig. 5:**
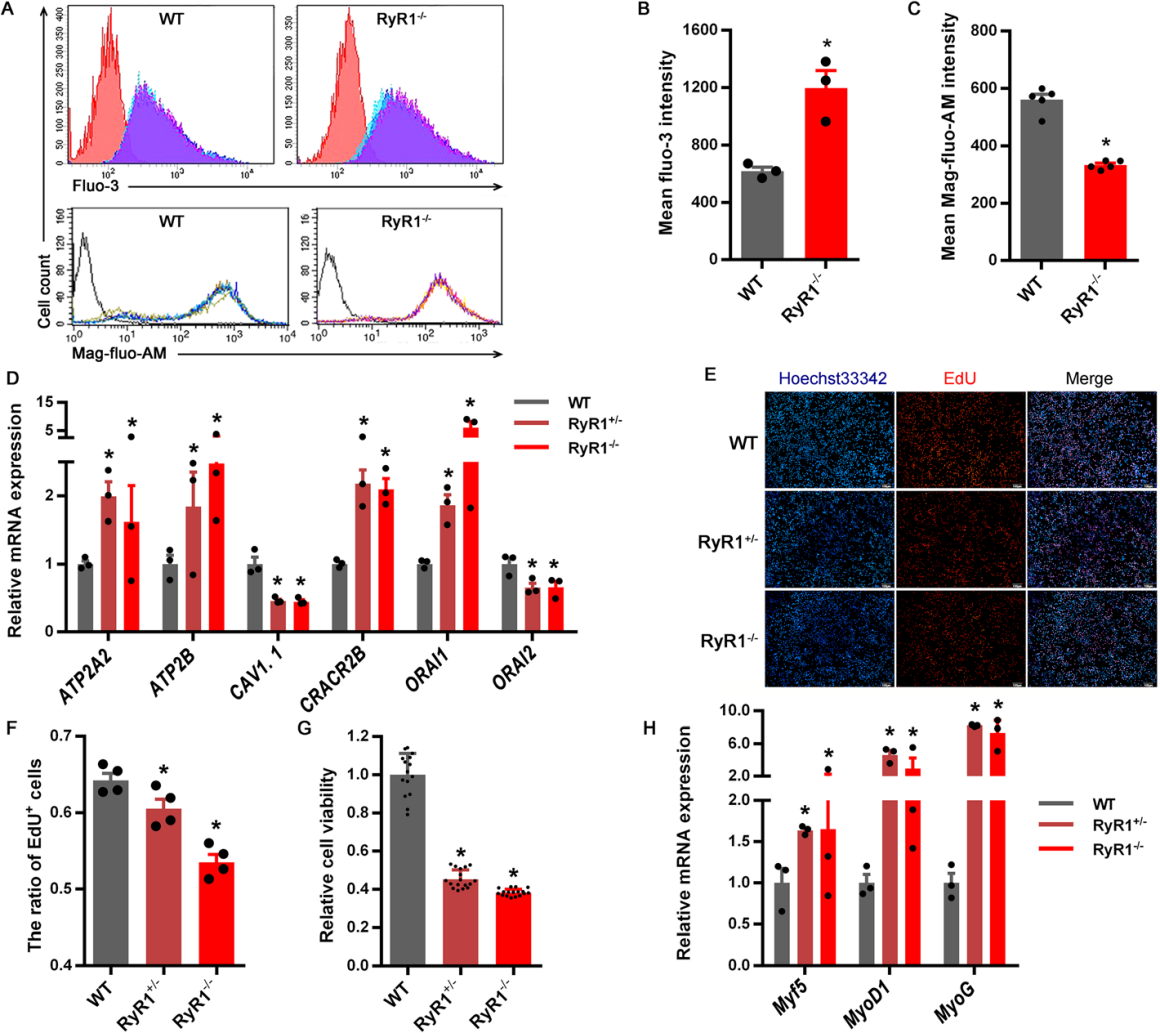
The effects of RyR1-knockout on cellular Ca^2+^ dynamics and differentiation of C2C12 myoblasts. **(A-C)** The concentration of Ca^2+^ in cytoplasm (labeled by Fluo-3, *n* = 3) and endoplasmic reticulum (labeled by Mag-fluo-AM, *n* = 5) of RyR1-KO cells. **(D)** The effect of RyR1-KO on the mRNA expression of Ca^2+^ channels (*n* = 3). **(E-G)** Proliferation and viability of RyR1-KO cells measured by EdU staining or CCK-8 test (*n* = 10). **(H)** Relative mRNA expression of *Myf5*, *MyoD1*, and *MyoG* in RyR1-KO cells (*n* = 3). **(I-J)** The expression of proteins related to MAPK signaling in RyR1-KO cells (*n* = 3). RyR1^−/−^ and RyR1^+/−^ represent homozygote and heterozygote of RyR1-knockout respectively. The data are presented as the mean ± SEM. *Represents significant difference between the two groups (*P* < 0.05)

RyR1 was successfully knockout by CRISPR/Cas-9 gene editing system targeting Exon 18 of *RyR1* via gRNA-3 which demonstrated the highest shearing efficiency among three designed gRNAs and resulted in the frame-shift mutation of *RyR1* (Fig. S3). Homozygote and heterozygote of RyR1-knockout cells, named as RyR1^−/−^ and RyR1^+/−^, were obtained by monoclonal cultivation and identified via gene sequencing on the target site and putative off-target sites of gRNA-3. Relative to the wild type cells (WT), RyR1^−/−^ showed the higher concentration of Ca^2+^ in the cytoplasm, but the lower level of Ca^2+^ in the ER (Fig. 5A-C). In RyR1^−/−^ or RyR1^+/−^, the mRNA expression of *ATP2A2*, *ATP2B*, *CRACR2B*, and *ORAI1* was significantly increased, while the mRNA expression of *CAV1.1* and *ORAI2* was decreased relative to WT (Fig. 5D). Cell proliferation (the proportion of EdU^+^ cells) of RyR1^−/−^ or RyR1^+/−^ significantly declined relative to WT (Fig. 5E and F), which was also demonstrated by the CCK-8 test (Fig. 5G). The mRNA expression of *Myf5*, *MyoD1*, and *MyoG* was significantly increased in RyR1^−/−^ or RyR1^+/−^ relative to WT (Fig. 5H).

### RyR1-knockout triggered apoptosis

**Fig. 6:**
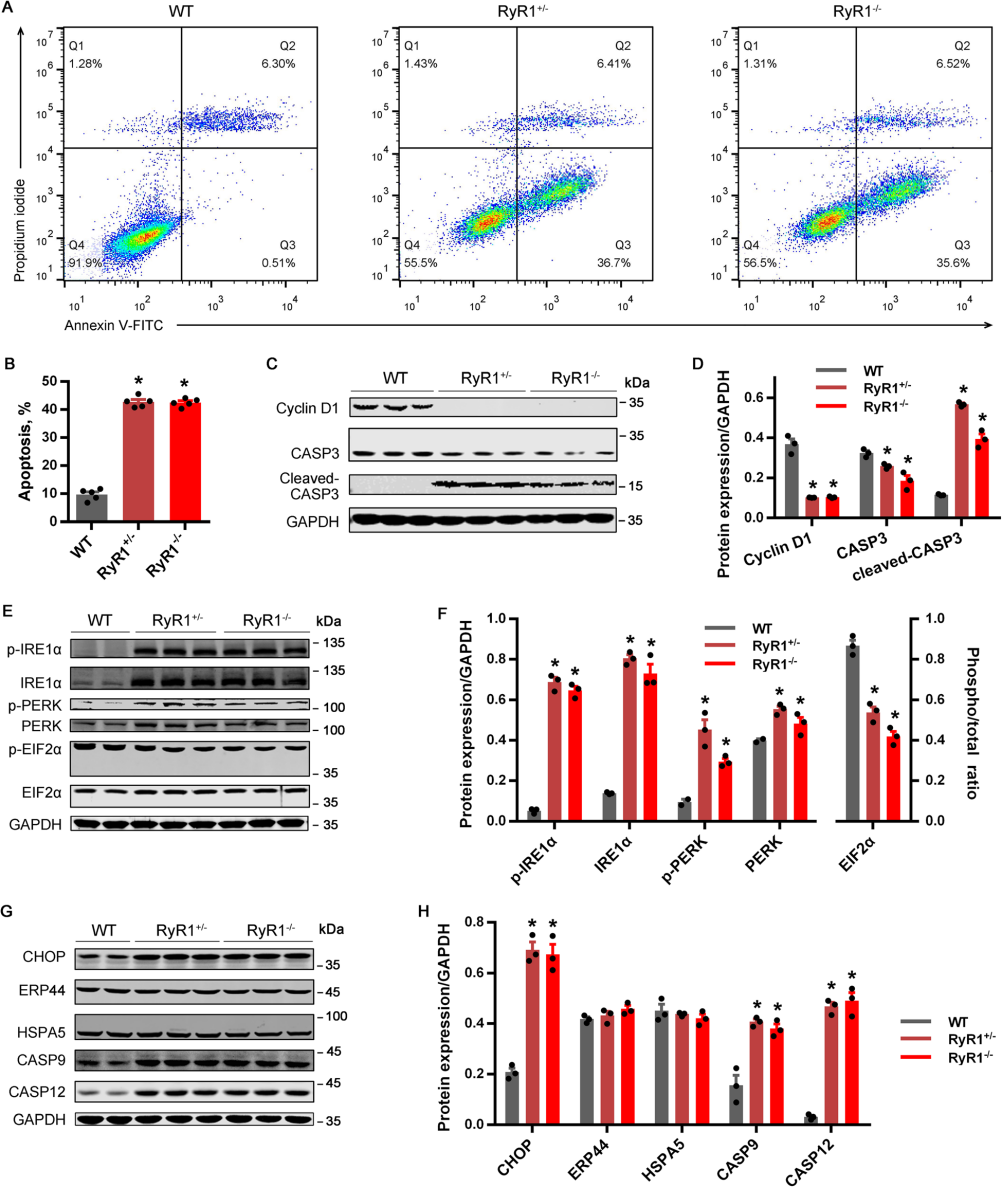
Endoplasmic reticulum stress-associated apoptosis in C2C12 cells was induced by RyR1-knockout (*n* = 3). **(A-B)** Apoptosis detection of RyR1-null cells through flow cytometry. **(C-D)** The protein expression of Cyclin D1, caspase-3 (CASP3), and cleaved-CASP3 in RyR1-KO cells. **(E-H)** The expression of proteins related to ER stress or ER stress-induced apoptosis in RyR1-KO cells. RyR1^−/−^ and RyR1^+/−^ represent homozygote and heterozygote of RyR1-knockout, respectively. The data are presented as the mean ± SEM. *Represents significant difference between the two groups (*P* < 0.05)

On d 2 during myogenic induction, apoptosis instead of myotube formation was significantly accelerated in both RyR1^−/−^ and RyR1^+/−^ relative to WT (Fig. 6A and B). The protein expression level of cyclin D1 and caspase-3 was significantly lowered in RyR1^−/−^ or RyR1^+/−^, but the expression of cleaved caspase-3 was significantly increased as compared with WT (Fig. 6C and D).

ER stress level of cells was evaluated, and both phosphorylated and total protein expression of IRE1α and PERK were dramatically elevated in RyR1^−/−^ and RyR1^+/−^, while phosphorylation of EIF2α was significantly decreased (Fig. 6E and F). As to several apoptosis-related proteins, the protein abundance of CHOP (also known as DDIT3), caspase-9, and caspase-12 in RyR1^−/−^ or RyR1^+/−^ was significantly increased relative to WT, while the protein expression of ERP44 and HSPA5 was not influenced (Fig. 6G and H).

## Discussion

Skeletal muscle mass is maintained by myogenic differentiation of myogenic progenitors and subsequent myoblast fusion [33], in which cytosolic Ca^2+^ dynamics plays a vital role [29]. Particularly, myotube formation requires net Ca^2+^ influx into myoblasts [34–36]. RyR1, serving as a major Ca^2+^ release channel of ER, has attracted the most intense research interests. However, the role of RyR1 in myogenesis remains unclear.

RyR1 protein expression pattern during myogenic differentiation of myoblastic C2C12 cells provides a well-controlled model for investigations of RyR1 function during myogenic differentiation [37]. In the current study, we observed that cytoplasmic Ca^2+^ concentration of C2C12 myoblasts was significantly elevated during myogenic differentiation, which was consistent with our previously study in primary myogenic cells of pigs [29]. Cytoplasmic Ca^2+^ dynamics is tightly regulated by various channels and transports in cells [38]. Relative to the extracellular matrix and ER, cytoplasmic Ca^2+^ concentration is maintained at very low levels (10–100 nmol/L) under resting conditions. Ca^2+^ is released from the ER, the main storage site of intracellular Ca^2+^, through the transmembrane channels RyR1 and ITPR1 [39, 40]. Cytoplasmic Ca^2+^ influx from extracellular matrix occurs through plasma membrane channels, such as CAV1, CAV2, and CAV3 [41]. In maintaining the resting state, excessive amounts of cytoplasmic Ca^2+^ re-accumulates in the ER by SR/ER Ca^2+^-ATPase (SERCA, also called ATP2A) [42] or is expulsed in the external milieu by plasma membrane Ca^2+^-ATPase (PMCA, also called ATP2B) [43, 44]. Furthermore, store-operated calcium entry (SOCE), mediated by STIM (ER Ca^2+^ sensors), activates CRAC and ORAI channels located at plasma membrane to maintain cellular Ca^2+^ homeostasis [45, 46].

It has been well known that lineage commitment and differentiation of myoblasts are governed by the programmed expression and functional activation of myogenic regulatory transcription factors (*MRFs*) [47, 48]. In this study, we discovered *RyR1* mRNA expression was increased more than 100-fold in C2C12 cells and almost 25-fold in myogenic cells of pigs during myogenic differentiation along with the normal expression pattern of Ca^2+^ transporters as well as *MRFs* during myogenic differentiation. In addition, the expression patterns of *RyR1* showed similar with *MyoG*, which was increased in the late stage of myogenesis. It strongly indicates that *RyR1* is not necessary for initial myogenic commitment, but more for later stage of myogenic differentiation.

Notably, RyR1 restriction via either DAN treatment or siRNA interference significantly decreased cytoplasmic Ca^2+^ concentration, and then blocked formation of multi-nuclei myotubes and expression of *MRFs*. As a major Ca^2+^ release channel of ER, the dramatic increase of RyR1 expression should be responsible for the significant elevation of cytosolic Ca^2+^ concentration. Therefore, we deduced that dramatic increase of RyR1 expression is required for the myoblast fusion at later stage of myogenesis. In addition, CAV1.1 is a physiological activator of RyR1 in the excitation–contraction coupling of skeletal muscle [49]. However, in this study, the expression of *CAV1.1* was not significantly increased as *RyR1* during myogenic differentiation, which indicated that *RyR1* expression in myogenic cells is independent of *CAV1.1*.

Thapsigargin, an inhibitor of SERCA, which mediated the reuptake of cytoplasmic Ca^2+^ into the sarcoplasmic reticulum, was used to increase cytoplasmic Ca^2+^ concentration. In the current study, the dose of THA was far below that used in previous studies [50, 51] in order to keep cell viability from the adverse effect of THA, and guaranteed that THA did not affect the efficiency of siRyR1-knockdown. As a result, THA treatment significantly increased cytoplasmic Ca^2+^ concentration but did not affect expression of myogenic specific genes. However, myotube formation was significantly inhibited by siRyR1-knockdown, but recovered by THA treatment, accompanied by the expression of *MRFs*. Therefore, RyR1-mediated elevation of cytoplasmic Ca^2+^ concentration was indispensable for myotubes formation.

To further explore the roles of RyR1 in myogenic differentiation and subsequent myoblast fusion, a model of RyR1-KO myoblasts by CRISPR/Cas9 gene-editing was employed in this study. The RyR1 Ca^2+^ release channel is composed of macromolecular complexes consisting of a homotetramer of 560-kDa RyR1 subunits that form scaffolds for proteins that regulate channel function including protein kinase A (PKA) and the phosphodiesterase PDE4D3 (both of which are targeted to the channel via the anchoring protein mAKAP), PP1 (targeted via spinophilin), and calstabin1 (FKBP12) [52, 53]. In contrast to the result of siRNA interference, we observed higher concentration of Ca^2+^ in the cytoplasm of RyR1-KO myoblasts, which suggested that considering the role of RyR1 as a calcium channel, RyR1-KO could differ from siRyR1 knockdown in terms of controlling Ca^2+^ dynamics between ER and cytoplasm. The deletion of RyR1 resulted in the dysfunction of the protein complex, losing the control of Ca^2+^ flowing out of the ER and accelerated Ca^2+^ flooding into the cytoplasm from the ER, which also distorted the expression patterns of other Ca^2+^ transporters. This observation is consistent with previous studies that demonstrated both over-activation and mutations of RyR1 resulted in leakage of Ca^2+^ from the SR [13, 54]. Cytoplasmic Ca^2+^ elevation directly activated ER stress [22, 23], and has a strong influence on differentiation through oxidative signaling and G0/G1 cell cycle arrest [55]. In the current study, expression of Cyclin D1 was abolished and cell viability was decreased in RyR1-KO cells, demonstrating that cell cycle and cell viability were suppressed during the enhanced ER stress caused by RyR1-KO mediated cytoplasmic Ca^2+^ elevation.

ER stress signaling gives rise to apoptosis [56]. In the present study, myogenic differentiation potential of RyR1-KO myoblasts reflected by *MRFs* expression was dramatically enhanced. However, the differentiation process of RyR1-KO myoblasts was interrupted by apoptosis, which indicated that RyR1 knockout make myoblasts too fragile to undertake the stress of myogenic induction. UPR was a protective response for cells under stress, however, excessive or prolonged UPR can cause apoptosis [57]. Caspase-3, belonging to a highly conserved family of cysteinyl aspartate-specific proteases, is an essential regulator of apoptosis. In the current study, cleaved activation of caspase-3 was significantly stimulated in RyR1-KO cells, which was supported by a previous study of RyRs-mediated ER stress [58]. In the present study, the total and phosphorylated protein expressions of IRE1α and PERK were sharply increased in RyR1-KO cells, indicating that RyR1-knockout activated serious ER stress. Furthermore, aggravated ER stress significantly increased expression of CHOP, caspase-9, and caspase-12. Meanwhile, the positive effects of ERP44 and HSPA5 against ER stress were not enhanced in RyR1-KO myoblasts. In addition, phosphorylation levels of JNK and Erk1/2, whose activations are beneficial for the resistance to ER stress-induced apoptosis [59, 60], were also significantly reduced in RyR1-KO cells. Therefore, we deduced that RyR1 deletion led to serious ER stress and excessive UPR than the tolerance thresholds of cells, and accounted for the apoptosis of RyR1-KO myoblasts upon myogenic induction.

In summary, dramatic increase in RyR1 expression is indispensable for myogenesis, and RyR1-mediated Ca^2+^ release plays a critical role in myoblast fusion at the later stage of myogenesis. RyR1-KO led to cytoplasmic Ca^2+^ elevation and enhanced myogenic differentiation potential, while serious ER stress and excessive UPR over the tolerance thresholds of cells resulted by RyR1-KO triggered the process of apoptosis of myoblasts upon myogenic induction. This study contributes to a novel understanding of the role of RyR1 in muscle development and related congenital myopathies, and provides a potential target for regulation of muscle characteristics and meat quality.

## Supplementary Information

**Additional file 1: Fig. S1** Cytoplasmic Ca^2+^ concentration of C2C12 cells during myogenic differentiation. **(A)** Immunostaining with myosin antibody on d 0, 2, 4, and 6 during myogenic differentiation. **(B)** Cytoplasmic Ca^2+^ signals of unfused C2C12 cells labeled by Fluo-3 on d 0–5 during myogenic differentiation (*n* = 3). **(C)** Quantitative results of cytoplasmic Ca^2+^ signals. **(D-E)** The proteins expression of RyR1 in C2C12 cells during myogenic differentiation (*n* = 3). GM: growth medium, representing cells cultured in growth medium before myogenic induction; DM: differentiation medium, representing cells on d 4 during myogenic differentiation cultured in differentiation medium. The data are presented as the mean ± SEM. *Represents significant difference with the value on d 0 (*P* < 0.05).

**Additional file 2: Fig. S2 (A)** The mRNA expression of *RyR3* during myogenic differentiation of C2C12 cells. *Represents significant difference with the value at D0 (*P* < 0.05). **(B)** The ratio of *RyR3* and *RyR1* mRNA expression in proliferating C2C12 cells. **(C)** The mRNA expression of *RyR1* and *RyR3* during myogenic differentiation of myogenic cells of pigs. **(D)** The ratio of *RyR3* and *RyR1* mRNA expression in proliferating myogenic cells of pigs. **(E)** The ratio of *RyR3* and *RyR1* mRNA expression of myogenic cells of pigs on d 4 during myogenic differentiation. *Represents significant difference between the two groups (*P* < 0.05). GM: growth medium, representing cells cultured in growth medium before myogenic induction; DM: differentiation medium, representing cells on d 4 during myogenic differentiation cultured in differentiation medium.

**Additional file 3: Fig. S3** The establishment of RyR1-knockout cells. **(A)** The plasmid vector used for CRISPR/Cas9 gene-editing. **(B)** The shearing efficiency of three gRNA targeted to *RyR1*. **(C)** The target site of the selected gRNA on *RyR1* gene. **(D)** Homozygote and heterozygote of RyR1-knockout, named as RyR1^−/−^ and RyR1^+/−^, was verified by gene sequencing.

**Additional file 4: Table S1** The primers used for qRT-PCR assays. **Table S2** Information of antibodies used for Western Blot. **Table S3** The potential off-target sites (OTS) of CRISPR/Cas9 system. **Table S4** Primers used for PCR and DNA sequencing.

## Data Availability

All data generated or analyzed during this study are included in this published article [and its supplementary information files].

## Abbreviations

ATP2A2: ATPase ER/SR Ca^2+^ transporting 2
ATP2B: ATPase plasma membrane Ca^2+^ transporting 1
Cas9: CRISPR associated protein 9
CASP3: Caspase-3
CAV1.1: Calcium voltage-gated channel subunit alpha1 S
CHOP: C/EBP homologous protein
CRACR2A: Calcium release activated channel regulator 2A
CRACR2B: Calcium release activated channel regulator 2B
CRISPR: Clustered Regularly Interspaced Short Palindromic Repeats
DAN: Dantrolene
DDIT3: DNA-damage inducible transcript 3
eIF2α: Eukaryotic translation initiation factor 2α
ER: Endoplasmic reticulum
Erk1/2: Extracellular regulated protein kinases
ERP44: ER protein 44
FKBP12: Calstabin1
FSC: Forward scatter
gRNA: Guide-RNA
GAPDH: Glyceraldehyde-3-phosphate dehydrogenase
HSPA5: Heat shock protein 5
ITPR1: Inositol 1,4,5-trisphosphate receptor type 1
JNK: Stress-activated protein kinase/Jun-amino-terminal kinase
MRFs: Myogenic regulatory transcription factors
Myf5: Myogenic factor 5
Mymk: Myomaker
MyoD1: Myogenic differentiation 1
MyoG: Myogenin
ORAI2: ORAI calcium release-activated calcium modulator 2
OTS: Off-target sites
PKA: Protein kinase A
RyR1: Ryanodine receptor 1
SOCE: Store-operated calcium entry
SR: Sarcoplasmic reticulum
STIM1: Stromal interaction molecule 1
TBST: Tris-Buffered saline and Tween-20
THA: Thapsigargin
UPR: Unfolded protein response
WT: Wild type
