# Supplementary figures and images for: Ryanodine receptor RyR1-mediated elevation of Ca2+ concentration is required for the late stage of myogenic differentiation and fusion
Source: J Anim Sci Biotechnol. 2022 Feb 11;13:9. doi: 10.1186/s40104-021-00668-x (PMC8832842; doi:10.1186/s40104-021-00668-x)

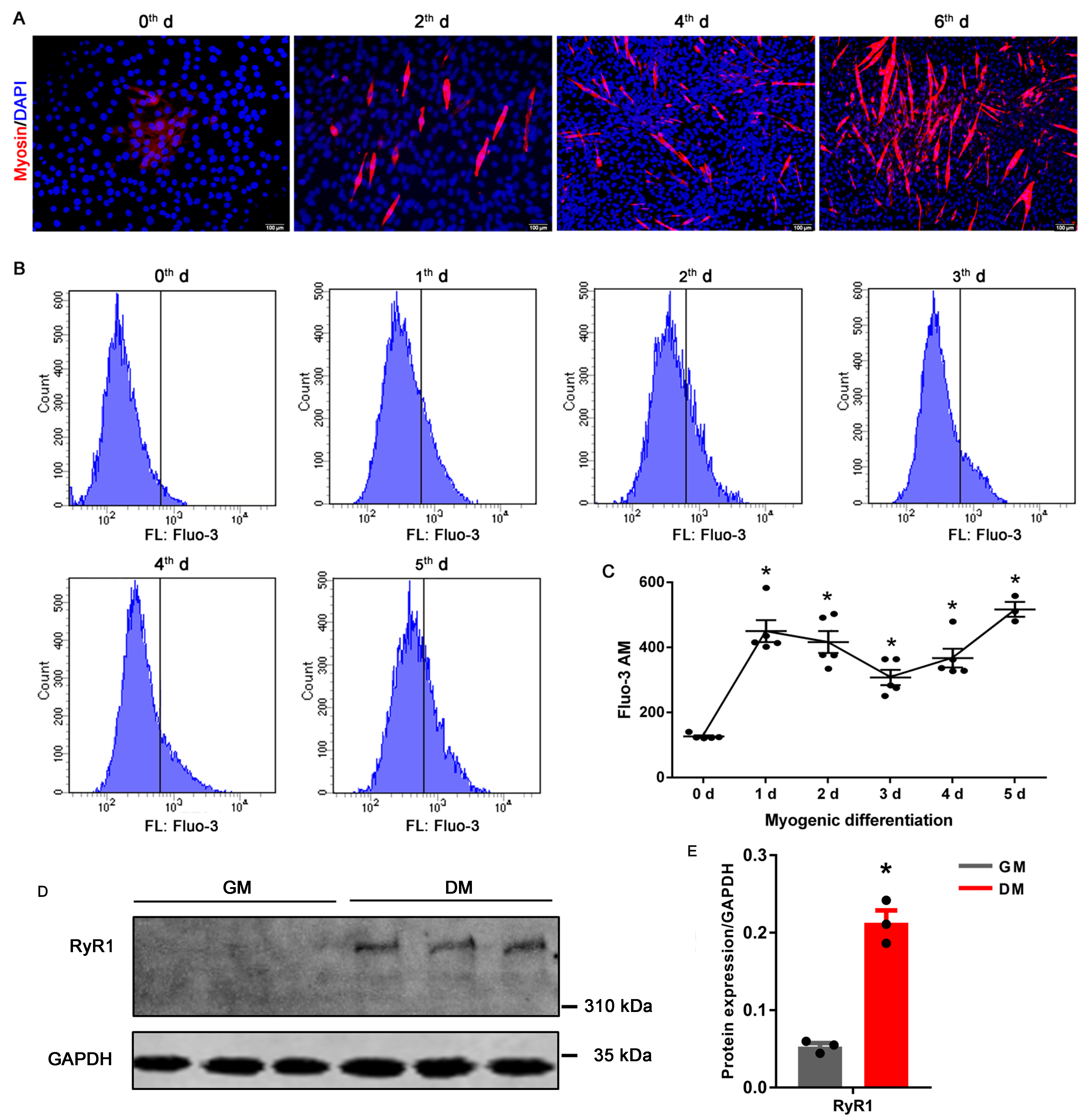

Supplement: Supplementary file 1 — Additional file 1: Fig. S1 Cytoplasmic Ca2+ concentration of C2C12 cells during myogenic differentiation. (A) Immunostaining with myosin antibody on d 0, 2, 4, and 6 during myogenic differentiation. (B) Cytoplasmic Ca2+ signals of unfused C2C12 cells labeled by Fluo-3 on d 0–5 during myogenic differentiation (n = 3). (C) Quantitative results of cytoplasmic Ca2+ signals. (D-E) The proteins expression of RyR1 in C2C12 cells during myogenic differentiation (n = 3). GM: growth medium, representing cells cultured in growth medium before myogenic induction; DM: differentiation medium, representing cells on d 4 during myogenic differentiation cultured in differentiation medium. The data are presented as the mean ± SEM. *Represents significant difference with the value on d 0 (P < 0.05). [file 40104_2021_668_MOESM1_ESM.tif]

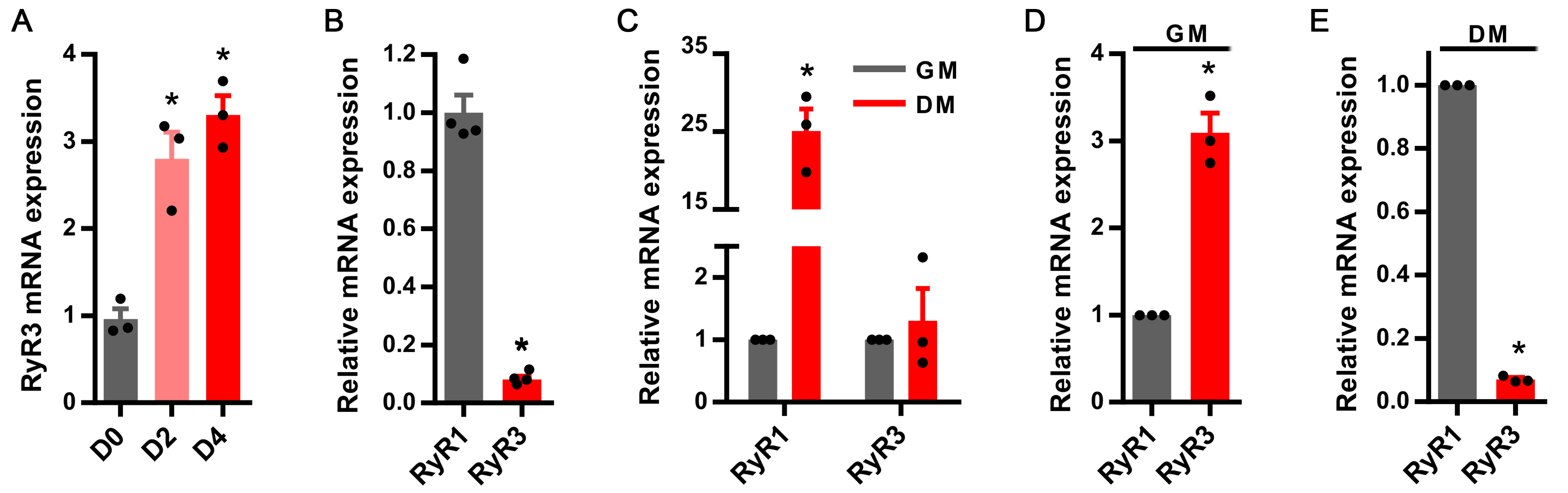

Supplement: Supplementary file 2 — Additional file 2: Fig. S2 (A) The mRNA expression of RyR3 during myogenic differentiation of C2C12 cells. *Represents significant difference with the value at D0 (P < 0.05). (B) The ratio of RyR3 and RyR1 mRNA expression in proliferating C2C12 cells. (C) The mRNA expression of RyR1 and RyR3 during myogenic differentiation of myogenic cells of pigs. (D) The ratio of RyR3 and RyR1 mRNA expression in proliferating myogenic cells of pigs. (E) The ratio of RyR3 and RyR1 mRNA expression of myogenic cells of pigs on d 4 during myogenic differentiation. *Represents significant difference between the two groups (P < 0.05). GM: growth medium, representing cells cultured in growth medium before myogenic induction; DM: differentiation medium, representing cells on d 4 during myogenic differentiation cultured in differentiation medium. [file 40104_2021_668_MOESM2_ESM.tif]

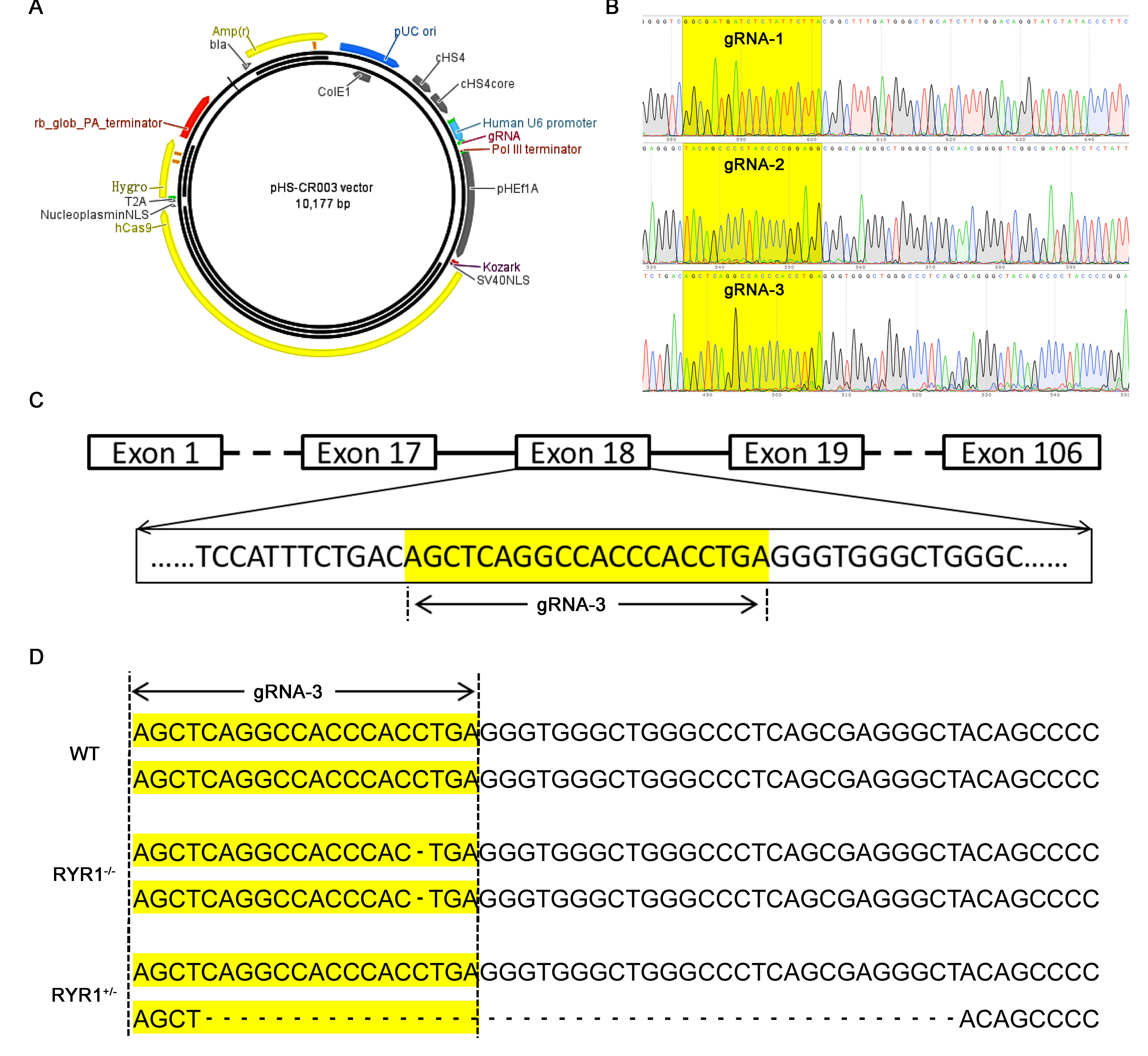

Supplement: Supplementary file 3 — Additional file 3: Fig. S3 The establishment of RyR1-knockout cells. (A) The plasmid vector used for CRISPR/Cas9 gene-editing. (B) The shearing efficiency of three gRNA targeted to RyR1. (C) The target site of the selected gRNA on RyR1 gene. (D) Homozygote and heterozygote of RyR1-knockout, named as RyR1−/− and RyR1+/−, was verified by gene sequencing. [file 40104_2021_668_MOESM3_ESM.tif]
