# Supplementary material for: Ryanodine receptor RyR1-mediated elevation of Ca2+ concentration is required for the late stage of myogenic differentiation and fusion
Source: J Anim Sci Biotechnol. 2022 Feb 11;13:9. doi: 10.1186/s40104-021-00668-x (PMC8832842; doi:10.1186/s40104-021-00668-x)
Supplement: Supplementary file 4 — Additional file 4: Table S1 The primers used for qRT-PCR assays. Table S2 Information of antibodies used for Western Blot. Table S3 The potential off-target sites (OTS) of CRISPR/Cas9 system. Table S4 Primers used for PCR and DNA sequencing. [file 40104_2021_668_MOESM4_ESM.docx]

**Table S1** The primers used for qRT-PCR assays.

| Gene name | Primer sequence | Annealing temperature (°C) | Product size (bp) |
| --- | --- | --- | --- |
| **Mus musculus** |  |  |  |
| *GAPDH* | F: 5' GTGTTCCTACCCCCAATGTG 3' | 60 | 349 |
|  | R: 5' CTTGCTCAGTGTCCTTGCTG 3' |  |  |
| *Myf5* | F: 5' AGACGCCTGAAGAAGGTCAA 3' | 60 | 308 |
|  | R: 5' GCAGCACATGCATTTGATACATC 3' |  |  |
| *MyoD1* | F: 5' GACCTGCGCTTTTTTGAGGACC 3' | 60 | 523 |
|  | R: 5' TGTAATCCATCATGCCATCAGA 3' |  |  |
| *MyoG* | F: 5' AAGTGAATGAGGCCTTCGAG 3' | 60 | 307 |
|  | R: 5' AGATTGTGGGCGTCTGTAGG 3' |  |  |
| *Mymk* | F: 5' ATCGCTACCAAGAGGCGTT 3' | 60 | 107 |
|  | R: 5' CACAGCACAGACAAACCAGG 3' |  |  |
| *RYR1* | F: 5' CCGCACCATCCTTTCATCTG 3' | 60 | 145 |
|  | R: 5' CTCGTCCTCATCTTCGCTCT 3' |  |  |
| *RYR3* | F: 5' GGTCGGGTGCATGATTAACC 3' | 62 | 176 |
|  | R: 5' CTGTCCCAAGATTCATGCGG 3' |  |  |
| *CAV1.1* | F: 5' ATGAGACTGGTCAAGCTGCT 3' | 60 | 189 |
|  | R: 5' GTTCCGGTTTATCTGCGTCC 3' |  |  |
| *ATP2B* | F: 5' TGCTGGAACTGATGTGGCTA 3' | 56 | 91 |
|  | R: 5' TCGTCCCCACATAACTGCTT 3' |  |  |
| *ATP2A2* | F: 5' ACCCAGACTTCGATGGAGTG 3' | 58 | 185 |
|  | R: 5' CATGGACAAGCAGATGGAGC 3' |  |  |
| *ORAI2* | F: 5' AACCTCAACTCCATCAGCGA 3' | 60 | 240 |
|  | R: 5' GACCACGAAGATGAGACCCA 3' |  |  |
| *CRACR2A* | F: 5' TATGACCTCACAGCCAAGCA 3' | 59 | 129 |
|  | R: 5' TTCCCGCTCCTTCTCATTGT 3' |  |  |
| *CRACR2B* | F: 5' AAGGGCTTCATCACTCGTCA 3' | 59 | 150 |
|  | R: 5' AAACTTCCCCAGGCCTAGAC 3' |  |  |
| *STIM1* | F: 5' TCAGGGAGTGGAACCAACTC 3' | 58 | 176 |
|  | R: 5' GGTAAGAGGAAGGCAGGTGT 3' |  |  |
| **Sus scrofa** |  |  |  |
| *GAPDH* | F: 5' TCGGAGTGAACGGATTTG 3' | 60 | 219 |
|  | R: 5' CCTGGAAGATGGTGATGG 3' |  |  |
| *RYR1* | F: 5' TTCCCTGTGTGTGTGCAATG 3' | 58 | 172 |
|  | R: 5' TTTGCTGTACTGTGTGGTGC 3' |  |  |
| *RYR3* | F: 5' AGCTGGAGTGGCAGTAACAT 3' | 58 | 211 |
|  | R: 5' CCATGCCTTCTATATCGCGC 3' |  |  |

**Table S2** Information of antibodies used for Western Blot.

| Antibodies | Catalog No. | Size (kDa) | Dilution | Company |
| --- | --- | --- | --- | --- |
| Phospho-S51 EIF2α | CSB-PA000518 | 36 | 1:500 | CSB, China |
| EIF2α | CSB-PA002295 | 36 | 1:500 | CSB, China |
| Phospho-S724 ERN1 | CSB-RA007795A724phHU | 110 | 1:500 | CSB, China |
| ERN1 | CSB-PA007795LA01HU | 110 | 1:500 | CSB, China |
| Phospho-T981 PERK | CSB-PA000746 | 130 | 1:500 | CSB, China |
| PERK | CSB-PA080010 | 130 | 1:500 | CSB, China |
| DDIT3 (CHOP) | CSB-PA006589LA01HU | 42 | 1:500 | CSB, China |
| ERP44 | CSB-PA580639 | 44 | 1:400 | CSB, China |
| HSPA5 | CSB-PA010827YA01MO | 73 | 1:500 | CSB, China |
| Cyclin D1 | #2922 | 36 | 1:1000 | CST, USA |
| Caspase-3 | #9662 | 35 | 1:1000 | CST, USA |
| Cleaved Caspase-3 (Asp175) | #9661 | 17/19 | 1:1000 | CST, USA |
| Caspase-9 | CSB-PA001235 | 46 | 1:500 | CSB, China |
| Caspase-12 | CSB-PA747641LA01HU | 39 | 1:500 | CSB, China |
| Phospho-p38 MAPK | #4511 | 43 | 1:10,000 | CST, USA |
| p38 MAPK | #8690 | 40 | 1:10,000 | CST, USA |
| Phospho-p44/42 MAPK (Erk1/2) (Thr202/Tyr204) | #4370 | 42/44 | 1:10,000 | CST, USA |
| p44/42 MAPK (Erk1/2) | #9194 | 42/44 | 1:10,000 | CST, USA |
| Phospho-SAPK/JNK (Thr183/Tyr185) | #4668 | 46/54 | 1:10,000 | CST, USA |
| SAPK/JNK | #9252 | 46/54 | 1:10,000 | CST, USA |
| GAPDH (D16H11) | #5174 | 37 | 1:10,000 | CST, USA |
| DyLight 800 Goat Anti-Rabbit IgG | 072-07-13-06 | - | 1:15,000 | KPL, USA |

**Table S3** The potential off-target sites (OTS) of CRISPR/Cas9 system.

| OTS | OTS sequence (5' to 3') | Chromosome | Position | Mismatchs |
| --- | --- | --- | --- | --- |
| OTS1 | 5` AGATCAAGCTACCCACCTGA 3` | Chr8 | 118347781 | 3 |
| OTS2 | 5` AGCTCAGGCCACCCACAGGT 3` | Chr3 | 143567607 | 3 |
| OTS3 | 5` ATCTCAGGTCACCCAACTGA 3` | Chr7 | 94640330 | 3 |
| OTS4 | 5` ACCTCAGGCCACACAGCTGA 3` | Chr7 | 125291005 | 3 |
| OTS5 | 5` AGCTCAGGGCAGACACCTGA 3` | Chr2 | 70832748 | 3 |
| OTS6 | 5` ACCTCAGGTCACCCACCTCA 3` | Chr2 | 117874706 | 3 |
| OTS7 | 5` AGCACAGGCCACCCAACTGG 3` | Chr19 | 25119551 | 3 |

**Table S4** Primers used for PCR and DNA sequencing.

| Name | Primer sequence | Tm (°C) | Product size (bp) |
| --- | --- | --- | --- |
| RYR1-KO | F: 5' TGCCCCTGTCTCTTCCTTCT 3' | 64.0 | 854 |
|  | R: 5' ACATTTGGGCAGTCTCCTGG 3' |  |  |
| OTS1 | F: 5' AGGAAGAAGAAGGTAGGCTAGGGT 3' | 62.0 | 745 |
|  | R: 5' AGCAGAGTAATGCCATGCCCA 3' |  |  |
| OTS2 | F: 5' AACAAACAATCCCTGAAAACAGGAG 3' | 58.0 | 523 |
|  | R: 5' CCAGAGGGATCTGGGGAATGA 3' |  |  |
| OTS3 | F: 5' ACTCATCACTGTGTAACTTCACCCT 3' | 58.0 | 620 |
|  | R: 5' CCCGTGCTGTTTCACAATAGACA 3' |  |  |
| OTS4 | F: 5' CAGTCAGTCGATGGAGCGGT 3' | 58.0 | 511 |
|  | R: 5' CATGACCCTATGGCCAGGTCC 3' |  |  |
| OTS5 | F: 5' ACTATCTTTCCCTAGCTGGCCT 3' | 62.0 | 700 |
|  | R: 5' TGCACTTCTAACCTATACCACATGC 3' |  |  |
| OTS6 | F: 5' GTATAAGAAGGGTAGAAACAGAGGAAGA 3' | 64.0 | 996 |
|  | R: 5' CCCTGAAGATTCCTATCGAGTG 3' |  |  |
| OTS7 | F: 5' CTGGACTATGGTTGTTGAGCTG 3' | 58.0 | 680 |
|  | R: 5' AATGTGGCGAGACCCCAC 3' |  |  |

^1^ RYR1-KO, DNA sequences covering target sites of CRISPR-Cas9-gRNA; OTS, Potential off-target sites of CRISPR/Cas9-gRNA.
